# Experimental Genetics of *Plasmodium berghei* NFU in the Apicoplast Iron-Sulfur Cluster Biogenesis Pathway

**DOI:** 10.1371/journal.pone.0067269

**Published:** 2013-06-21

**Authors:** Joana M. Haussig, Kai Matuschewski, Taco W. A. Kooij

**Affiliations:** Parasitology Unit, Max Planck Institute for Infection Biology, Berlin, Germany; University of Melbourne, Australia

## Abstract

Eukaryotic pathogens of the phylum *Apicomplexa* contain a non-photosynthetic plastid, termed apicoplast. Within this organelle distinct iron-sulfur [Fe-S] cluster proteins are likely central to biosynthesis pathways, including generation of isoprenoids and lipoic acid. Here, we targeted a nuclear-encoded component of the apicoplast [Fe-S] cluster biosynthesis pathway by experimental genetics in the murine malaria parasite *Plasmodium berghei*. We show that ablation of the gene encoding a nitrogen fixation factor U (NifU)-like domain containing protein (*NFUapi*) resulted in parasites that were able to complete the entire life cycle indicating redundant or non-essential functions. *nfu* ^–^ parasites displayed reduced merosome formation *in vitro*, suggesting that apicoplast NFUapi plays an auxiliary role in establishing a blood stage infection. NFUapi fused to a combined fluorescent protein-epitope tag delineates the *Plasmodium* apicoplast and was tested to revisit inhibition of liver stage development by azithromycin and fosmidomycin. We show that the branched apicoplast signal is entirely abolished by azithromycin treatment, while fosmidomycin had no effect on apicoplast morphology. In conclusion, our experimental genetics analysis supports specialized and/or redundant role(s) for NFUapi in the [Fe-S] cluster biosynthesis pathway in the apicoplast of a malarial parasite.

## Introduction

Iron-sulfur [Fe-S] clusters are inorganic cofactors that constitute one of the most ancient and ubiquitous prosthetic groups. Proteins containing [Fe-S] clusters have diverse functions [1], [2]. Arguably, the most prominent function is in electron transport and the generation of ATP for the cell’s energy requirements. The biosynthetic pathway leading to [Fe-S] clusters is complex and involves numerous components [1]–[4]. In eukaryotes, different biogenesis machineries have been identified that assemble [Fe-S] clusters in various cellular compartments, namely the cytoplasmic iron-sulfur protein assembly (CIA) [5], the mitochondrial iron-sulfur cluster (ISC) [6], and the plastid-localized sulfur utilization factor (SUF) [7], [8] systems. Furthermore, the nitrogen fixation (NIF) system, which has been proposed to be the [Fe-S] cluster biosynthesis pathway to have evolved earliest [4], was first discovered in the Gram-negative bacterium *Azotobacter vinelandii* and functions in the maturation of nitrogenase [9]. In spite of the different systems in bacteria and eukaryotes, the basic principles of [Fe-S] cluster biogenesis are conserved. First, the [Fe-S] cluster is assembled *de novo* on a scaffold protein. Then, the [Fe-S] cluster is transferred from the scaffold protein to a target apoprotein and assembled into the polypeptide chain.

**Figure 1:**
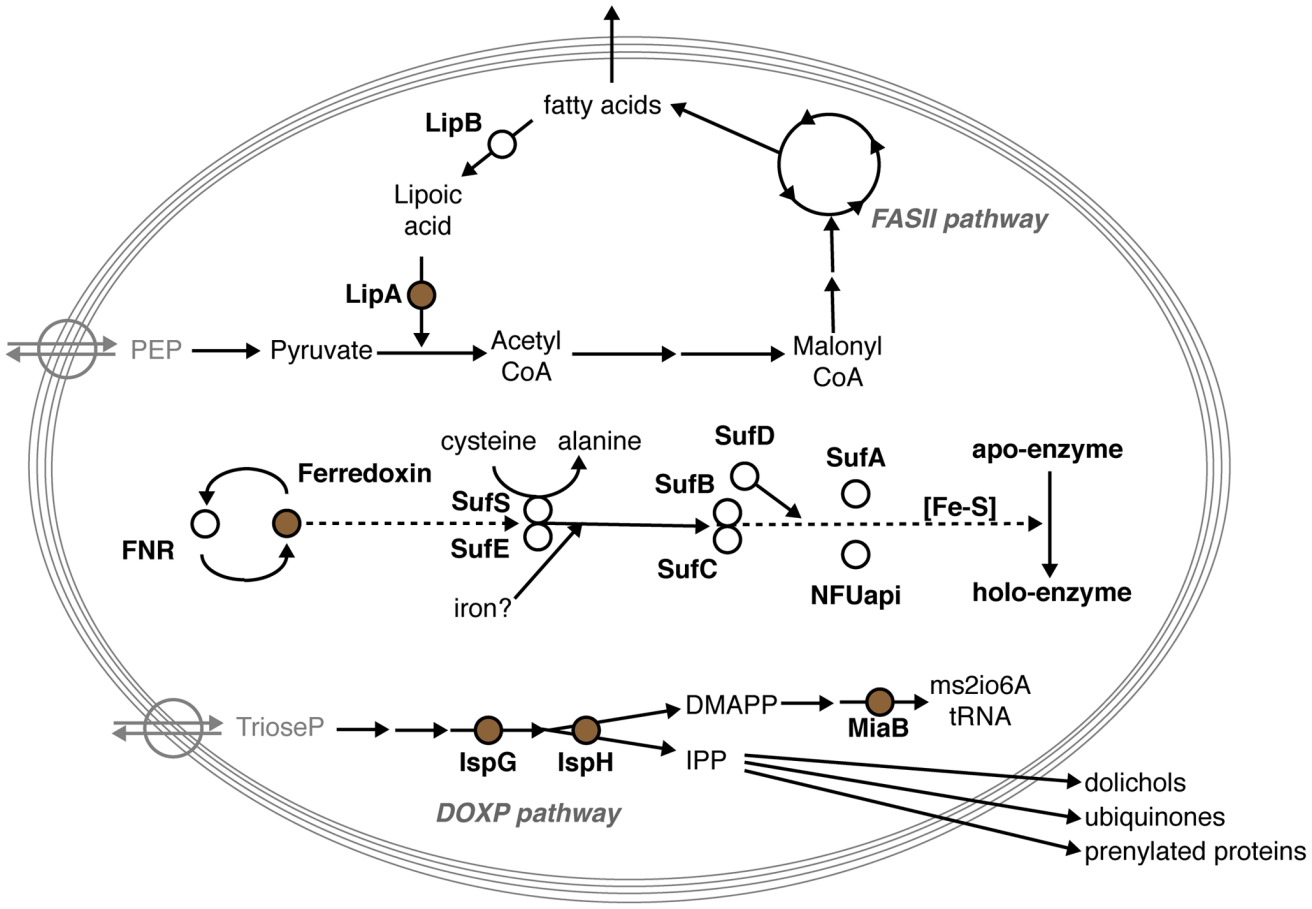
Overview of *Plasmodium berghei* apicoplast-resident proteins containing a [Fe-S] cluster or involved in the [Fe-S] cluster biosynthesis pathway. Shown is an overview of the principal apicoplast localized biosynthesis pathways: fatty acid synthesis (FASII pathway), non-mevalonate isoprenoid synthesis (DOXP pathway), and [Fe-S] cluster synthesis (center), with their respective precursors and products. The five predicted apicoplast [Fe-S] cluster containing proteins are depicted in brown.

Though [Fe-S] cluster containing and generating proteins have not been studied as intensively in apicomplexan parasites as in some other systems, they already received considerable attention [4], [8], [10], [11]. *Plasmodium* species harbor genes that could be involved in all three [Fe-S] cluster biosynthetic pathways found in eukaryotes. Orthologs encoding most components involved in the mitochondrial ISC system, important for citric acid cycle, mitochondrial electron flow, and biogenesis of cytochrome oxidase, were readily identified. In contrast, only two genes with putative cytoplasmic roles in the CIA machinery were found. Finally, malaria parasites are equipped with components of the SUF system, which are predicted to target to the vestigial plastid unique to this phylum of obligate intracellular parasites. Indeed, immunofluorescence data have confirmed the targeting of *Plasmodium falciparum* SUFC to this organelle, the so-called apicoplast [12]. [Fe-S] cluster-containing proteins in the apicoplast are central to several biosynthesis pathways, including mevalonate-independent isoprenoid biosynthesis, lipoic acid metabolism, and biogenesis of [Fe-S] clusters itself (Fig. 1; Table 1). And yet, no phenotypical analyses of any experimentally modified apicomplexan parasite are available that indicate either essential, distinct stage-specific, or dispensable roles for any component of the [Fe-S] cluster biogenesis pathway in the apicoplast.

**Table 1:** *Plasmodium* [Fe-S] biosynthesis pathway proteins of the apicoplast.

Gene name	Predicted function	*P. berghei* a	*P. falciparum* a	ApicoAP^b^	PlasmoAP^b^	PlasMit^b^	MitoProtII^b^
*SUFA*	[Fe–S] cluster transfer protein	PBANKA_123740	PF3D7_0522700	ApicoTP	++/++	non-mito (99%)	0.9862
*SUFB*	Sulfur mobilization scaffold protein	PBANKA_API0012	PFC10_API0012	–	–	–	–
*SUFC*	Sulfur mobilization scaffold protein	PBANKA_102920	PF3D7_1413500	No SP	0/++	mito (91%)	0.5104
*SUFD*	Sulfur mobilization, complexed withSUFB & C	PBANKA_094350	PF3D7_1103400	ApicoTP	++/++	non-mito (99%)	0.5501
*SUFE*	Desulfurase activator and sulfide“transferase”	PBANKA_030380	PF3D7_0206100	ApicoTP	++/++	non-mito (99%)	0.9182
*SUFS*	Cysteine desulfurase	PBANKA_061430	PF3D7_0716600	ApicoTP	++/++	non-mito (99%)	0.2619
*NFUapi*	NifU-like scaffold protein	PBANKA_082230	PF3D7_0921400	ApicoTP	++/++	non-mito (99%)	0.2084

a Gene IDs of the *P. berghei* and *P. falciparum* orthologs (http://PlasmoDB.org).

b Putative targeting of the *P. falciparum* SUF pathway proteins to the apicoplast or mitochondrion was predicted using four different algorithms. ApicoAP [35] predicts whether a given protein lacks the required signal peptide (“No SP”), contains a signal peptide but no transit peptide (“non-ApicoTP”), or is an apicoplast targeted protein (“ApicoTP”) that uses the bipartite signaling mechanism. PlasmoAP [36] indicates the likelihood of the presence of the required signal peptide followed by the likelihood of an apicoplast localization (“-” = unlikely, “0″ = undecided, “+” = likely, “++” = very likely). PlasMit [37] predicts the likelihood of a mitochondrial localization for *P. falciparum* proteins (“non-mito (99%)”, “mito (91%)”, and “mito (99%)”). MitoProtII [38] gives a probability score for the likelihood of mitochondrial localization but is not optimized *Plasmodium* sequences. Note that no analysis was done for *SUFB* as the gene is encoded on the apicoplast genome and hence needs no targeting sequences.

Here, we present an experimental genetics analysis of the NifU-like domain containing protein (NFU) in the [Fe-S] biosynthesis pathway of the *Plasmodium berghei* apicoplast. We verify localization of NFU to the apicoplast and demonstrate that it is dispensable for life cycle progression, though our data suggest an auxiliary function in liver stage maturation, at least in *in vitro* merosome formation.

## Results

### Selection of NFU in the Apicoplast [Fe-S] Cluster Pathway as Target Gene

We first reanalyzed the predicted targeting sequences and putative subcellular localization of the predicted apicoplast-localized SUF system components. This revealed that not all predictions were conclusive, *i.e.* SUFC misses a signal peptide sequence, indicating that some revisions might be required with the occurrence of new experimental data and optimized prediction tools. We were able to confirm the presence of a complete set of genes involved in the plastid SUF system in *Plasmodium* species (Fig. 1 and Table 1).

**Figure 2:**
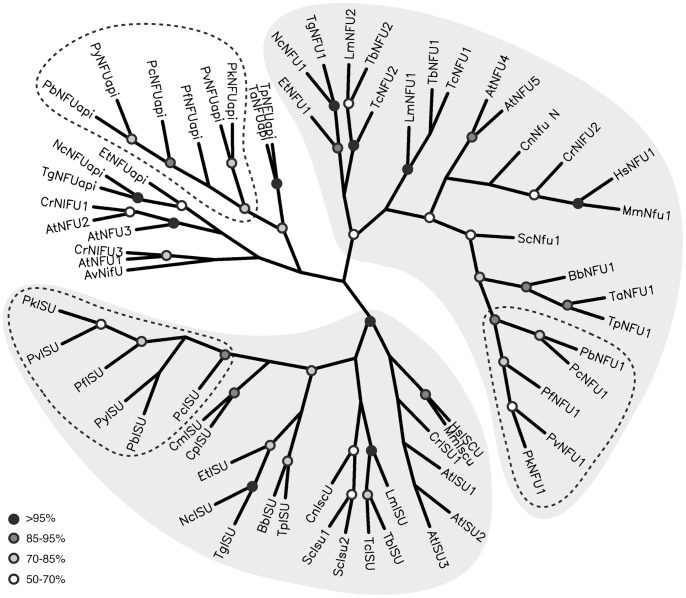
Phylogenetic analysis of apicomplexan and other representative eukaryotic NifU-like domain containing proteins. Maximum likelihood distances were calculated for 68 NifU-like domain containing proteins, including all sequences identified for apicomplexan parasites and two representative species from the plant, fungus, and animal kingdoms (see also Table S1 in File S1). Circles represent branch points with bootstrap values of 95–100% (black), 85–95% (dark gray), 70–85% (light gray), and 50–70% (white). *Azotobacter vinelandii* NifU was used as the outgroup. The mitochondrial “ISU” and “NFU1” clades (light gray shading) constitute of proteins involved in the ISC system and were well supported in all different analyses with bootstrap values of 100% in all cases for “ISU” and 52–78% for “NFU1”. The remaining “NFU” sequences, constituting members of the SUF system restricted to plants, algae, and apicomplexan species, group in clades with sequences from the same or related species, but do not consistently form a plastid “NFU” clade. The *Plasmodium* subclades are indicated by dashed lines. Note that the apicomplexan parasites of the genus *Cryptosporidium* that are known to lack an apicoplast and fully functional mitochondrion [44] lack both genes encoding NFU-like proteins.

Malaria parasite genomes encode three NifU-like domain containing proteins, related to the bacterial NIF system. Two of these were predicted to target to the mitochondrion, one ISU/IscU ortholog (PBANKA_131820) and one NFU1/NfuA ortholog (PBANKA_083170). The third protein, that we termed NFUapi, is expected to localize to the apicoplast (PBANKA_082230). Alignments of the predicted apicoplast-targeted NifU-like domain containing protein (NFUapi), revealed conservation within the genus *Plasmodium* but not when compared with other *Apicomplexa* (Fig. S1 in File S1). Phylogenetic analyses of the NifU-like domains confirmed the separation of *Plasmodium* NifU-like domain containing proteins into three groups. The ISU/IscU and NFU1/NfuA orthologs predicted to target to mitochondria consistently formed clades with orthologs from a variety of eukaryotic species (Fig. 2 and Table S1 in File S1). As expected from the limited sequence conservation of the predicted apicoplast targeted NFU, these analyses did not support a plastid-specific clade of apicomplexan and plant NFU sequences.

### NFUapi Localizes to the *Plasmodium* Apicoplast

**Figure 3:**
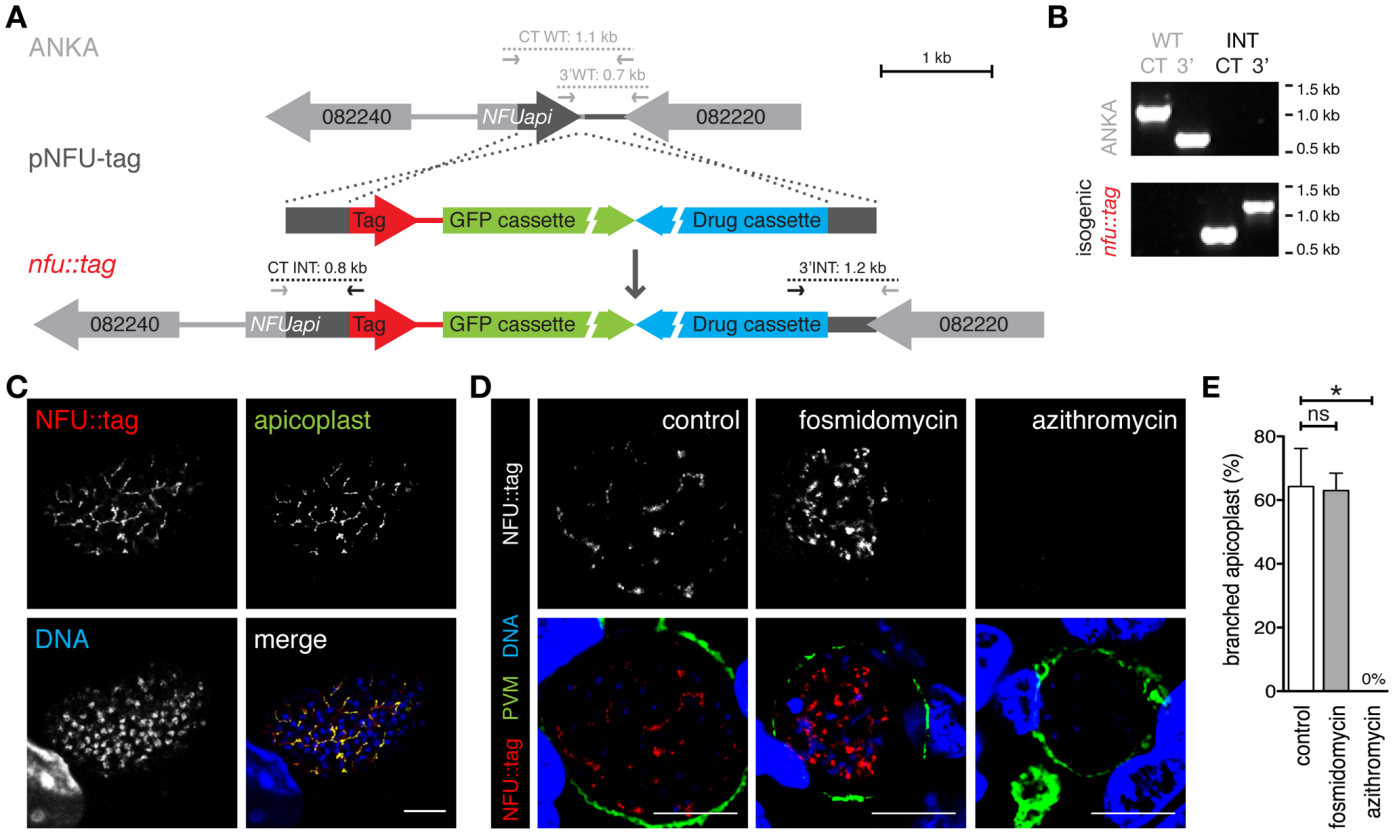
*Plasmodium berghei* NFUapi localizes to the apicoplast. (A) Replacement strategy to generate stable parasite lines that express the endogenous *NFUapi* fused to the mCherry-3xMyc tag (red). In addition, recombinant parasites contain the high-expressing GFP cassette (green) and the drug-selectable *hDHFR-yFcu* cassette (blue). Integration-specific (CT-INT and 3′INT) and wild type-specific (CT-WT and 3′WT) primer combinations (Table S2 in File S1) are indicated by arrows and expected fragments as dotted lines. (B) PCR-based genotyping of *nfu::tag* parasites to verify successful fusion of *NFUapi* with the mCherry-3xMyc tag. Absence of WT signals confirms the purity of the isogenic parasite line. (C) Co-staining of fixed, *nfu::tag* parasite-infected hepatoma cells 48 hours after sporozoite infection using anti-myc and anti-ACP antibodies. Note substantial overlap between NFUapi and the signature apicoplast protein. Bar, 10 ìm. (D) Drug treatment of *nfu::tag*-infected hepatoma cells to corroborate apicoplast localization of NFUapi. During liver stage development *nfu::tag*-infected cells were left untreated (control), treated with 100 ìM fosmidomycin, or 1 ìM azithromycin. Liver stages were stained with anti-myc antibodies and anti-sera against upregulated in infective sporozoite protein 4 (UIS4), a signature protein of the parasitophorous vacuolar membrane (PVM). Bars, 10 ìm. (E) Quantification of the percentage of *nfu::tag* liver stages from panel (D) with branched apicoplasts (control, n = 375; 100 ìM fosmidomycin, n = 375; 1 ìM azithromycin, n = 339). Shown are mean percentages of four independent experiments (± S.D.). ns, non-significant; *, *P*<0.05 (Non-parametric, two-tailed Mann-Whitney’s test).

To verify the predicted apicoplast targeting of NFUapi, we generated a transgenic parasite line that expresses NFUapi with a combined fluorescent protein-epitope tag (Fig. 3A). Isogenic parasites expressing a NFU::tag fusion protein were readily obtained (Fig. 3B). Live parasites only produced faint undefined signals in developing midgut-associated oocysts and cultured liver stage parasites (data not shown). To enhance the NFU::tag signal, we stained fixed liver stage parasites at 48 hours after infection with antibodies against the myc epitope tag (Fig. 3C). The extended branched structure is reminiscent of the apicoplast. Co-localization with a signature apicoplast protein, acyl carrier protein (ACP [13]), supported this notion (Fig. 3C). Using immunofluorescence imaging, we could detect the branched apicoplast by staining with anti-myc antibodies in >60% of all late liver stage parasites (Fig. 3E).

Because of the close association of mitochondrion and apicoplast in apicomplexan parasites, additional confidence into *bona fide* apicoplast targeting can be obtained by antibiotic treatment, resulting in specific disintegration of the apicoplast. Accordingly, *nfu::tag* liver stage parasites were treated with the azalide antibiotic azithromycin (Fig. 3D and E). Apicoplast disintegration and a complete loss of NFUapi signal corroborate the notion that NFUapi localizes to the apicoplast.

During the course of these experiments, we also re-visited an important, yet controversial, topic, *i.e.* whether the phosphonic acid antibiotic fosmidomycin exerts causal-prophylactic activity against *Plasmodium* parasites [14], [15]. When parasites were treated with a high dose (100 µM) of fosmidomycin, the NFUapi signal showed a branched apicoplast in 63% of infected hepatoma cells, similar to untreated *nfu::tag*-infected hepatoma cells (Fig. 3D and E).

We conclude that, as predicted (Table 1), NFUapi localizes to the *Plasmodium* apicoplast.

### Dispensable and Auxiliary Roles for *PbNFUapi*

**Figure 4:**
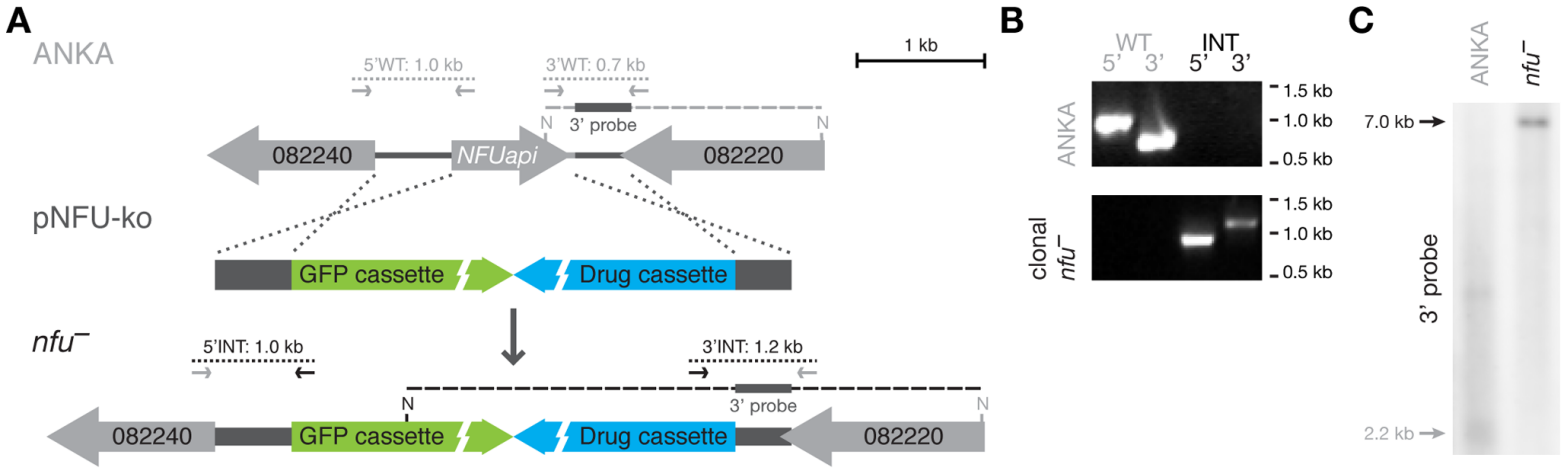
Targeted deletion of *Plasmodium berghei NFUapi*. (A) Replacement strategy to delete *PbNFUapi*. The endogenous *NFUapi* gene (gray arrow) was targeted with a replacement plasmid (pNFU-ko) containing 5′ and 3′ regions (dark gray bars) flanking the open reading frame (light gray arrow), a high-expressing GFP cassette (green), and the *hDHFR-yFcu* drug-selectable cassette (blue). Integration- and WT-specific primer combinations (Table S2 in File S1) and expected fragments are indicated. (B) PCR-based genotyping of *nfu^–^* parasites to verify successful deletion of *NFUapi*. Absence of WT-specific signals in the clonal *nfu^–^* line confirms purity of the knockout parasites. (C) Southern blot analysis of the clonal *nfu* ^–^ parasite line shows bands of the expected sizes (arrows) in NdeI restriction-digested gDNA of WT (gray, 2.2 kb) and recombinant parasites (black, 7.0 kb). The 3′ homologous sequence used for targeted integration of the transfection vectors (dark gray bar in [A]) was used as the probe.

In order to explore the role of *NFUapi*, we next targeted this gene by a genetic replacement strategy (Fig. 4). *NFUapi* was readily deleted by double homologous/ends-out recombination (Fig. 4A) and a clonal *nfu* ^–^ parasite line devoid of WT contamination was generated by intravenous injection of limiting dilutions of parasites into mice (Fig. 4B and C).

**Figure 5:**
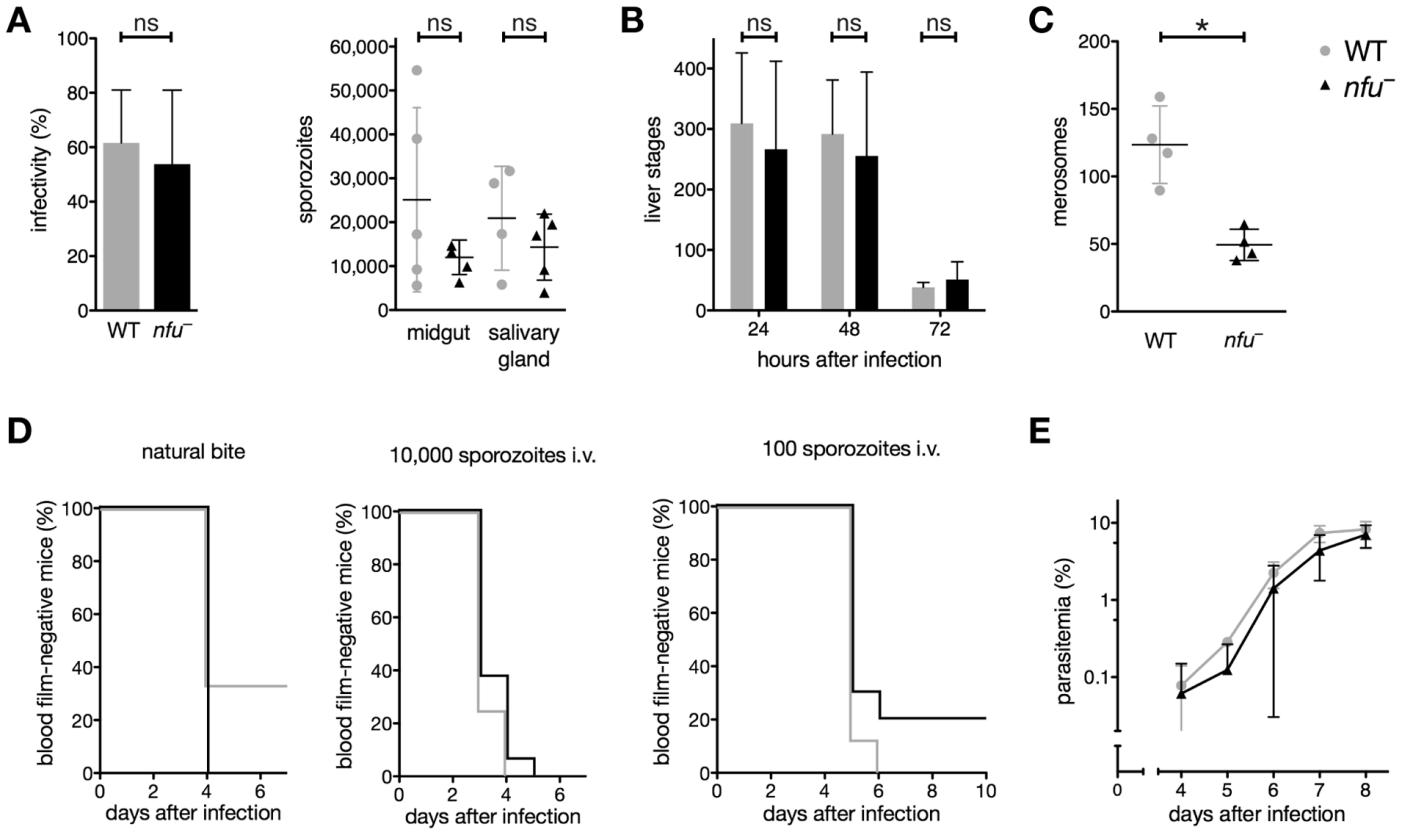
*NFUapi* is dispensable for life cycle progression *in vivo*, yet might exert auxiliary function(s) during merosome formation *in vitro*. (A) Infectivity and mean sporozoite numbers (± S.D.) in midgut-associated oocysts (day 14 after infection) and salivary glands (day 17–21 after infection) of WT and *nfu^–^*-infected mosquitoes from five independent feeding experiments did not differ significantly (*P*>0.05; non-parametric, two-tailed Mann-Whitney’s test). (B) Liver stage development of *nfu* ^–^ parasites in cultured hepatoma cells. Shown are mean numbers (± S.D.) 24, 48, and 72 hours after infection from four independent experiments (three replicates each). WT and *nfu* ^–^ parasites numbers did not differ significantly over the time course or at individual time points (*P*>0.05; two-way ANOVA followed by Bonferroni posttests). (C) Merosome formation at 72 hours after inoculation of cultured hepatoma cells with 10,000 sporozoites. Shown are mean values of four independent experiments (± S.D.). Merosome formation of *nfu* ^–^ liver stage parasites was significantly reduced compared with WT (*P*<0.05, non-parametric, two-tailed Mann-Whitney’s test). (D) Kaplan-Meier analysis of time to malaria blood stage infection. C57BL/6 mice were infected with WT (gray) or *nfu* ^–^ (black) parasites by natural bite by 5–7 infected mosquitoes (single experiment; WT, n = 3; *nfu* ^–^, n = 7), or by intravenous injection of 10,000 (three independent experiments; WT, n = 8; *nfu* ^–^, n = 16) or 100 (two independent experiments; WT, n = 8; *nfu* ^–^, n = 10) isolated sporozoites. Animals were monitored daily for presence of parasites in Giemsa-stained thin blood smears. Survival curves of WT and *nfu* ^–^ did not differ significantly (*P*>0.05 for both Mantel-Cox and Gehan-Breslow-Wilcoxon tests). (E) Asexual blood stage development following intravenous injection of 1,000 infected erythrocytes. Parasitemia of recipient mice (n = 10, from three independent experiments) was monitored daily by examination of Giemsa-stained thin blood smears. Shown are mean values (± S.D.). WT and *nfu* ^–^ parasites blood stage development did not differ significantly over the time course or at individual time points (*P*>0.05; two-way ANOVA followed by Bonferroni posttests).

Successful generation of *nfu* ^–^ parasites permitted detailed *in vivo* phenotyping of parasite fitness during life cycle progression. We first tested transmission to the *Anopheles* vector and sporogony (Fig. 5A). Mosquito infectivity, numbers of mosquito midgut- and salivary gland-associated sporozoites of *nfu^−^* parasites were within WT range. Next, we isolated sporozoites to infect cultured hepatoma cells (Fig. 5B). Numbers of *nfu* ^–^ liver stage parasites were similar to those of WT parasites. When we quantified merosomes, merozoite-filled vesicles budding from mature liver stage parasites [16], we noticed a significant (*P*<0.05) reduction of ∼60% as compared to WT parasites (Fig. 5C).

This finding prompted us to monitor the time to detectable blood stages upon sporozoite infection *in vivo*, the so-called prepatent period (Fig. 5D). We tested prepatency by either intravenous injection of 100 or 10,000 sporozoites or by exposure to bites of 5–7 infected *Anopheles stephensi* mosquitoes. Irrespective of the mode of sporozoite delivery, initiation of blood infection was comparable between *nfu^–^* and WT-infected animals (Fig. 5D). None of the minor differences reached statistical significance, which led us to conclude that *NFUapi* does not play an important role in the initiation of a blood stage infection.

Finally, we infected naïve NMRI mice by transfusion of 1,000 infected erythrocytes (Fig. 5E). Mice infected with *nfu* ^–^ parasites displayed a small, but non-significant, delay in blood stage expansion. After 8 days this difference was completely abrogated and all *nfu* ^–^-infected animals displayed high parasitemia. Together, our phenotyping established a dispensable role of *NFUapi* for life cycle progression of the malaria parasite, at least *in vivo*, though *NFUapi* might exert auxiliary function(s) during merosome formation as indicated by our *in vitro* analysis.

## Discussion

[Fe-S] cluster biogenesis has been well studied in yeast and bacteria, and to a lesser extent in higher eukaryotes [1]–[4]. Very little functional data are yet available for apicomplexan parasites. A recent biochemical study has focused on the *P. falciparum* plastid SUF system and provided convincing evidence that SUFC is localized to the apicoplast [12]. Both in bacteria and plants, SUFC interacts with SUFB [17]–[19], which was confirmed in *P. falciparum* [12]. Furthermore, ATPase activity of recombinant *Pf*SUFB and *Pf*SUFC proteins provided intriguing evidence for the evolutionary conservation of the plastid SUF system between plants and apicomplexan parasites. In *Arabidopsis thaliana*, SUFB and SUFC display ATPase activity [19], which contrast with the bacterial SufB that lacks this activity.

A recent publication favors the role of *Escherichia coli* NfuA as a [Fe-S] cluster carrier, rather than a scaffold protein, acting downstream of both the SUF and ISC systems [20] (Fig. 1). Likewise, *E. coli* SufA, initially suggested to act as a scaffold for [Fe-S], acts as a shuttle transferring [Fe-S] clusters from scaffold proteins to apoproteins [21]–[23]. The only available functional data for NifU-like domain containing proteins in apicomplexan parasites to date is the confirmation of the apicoplast subcellular localizations of *Toxoplasma gondii* NFUapi (TGME49_021920) [24]. Furthermore, Sheiner *et al.* were apparently able to delete *TgNFUapi* by experimental genetics, because the authors listed the gene as non-essential [24]. The *P. berghei* malaria model allows in-depth analysis of the entire life cycle including transmissions from the vertebrate host through sexual development in the mosquito vector and back to vertebrate hosts, where clinical symptoms are manifest. We have verified the apicoplast localization of *P. berghei* NFUapi and loss-of-function mutants were readily obtained *in vivo*. A careful analysis of life cycle progression revealed that *PbNFUapi* is not critical for any step in stage conversion, host switch, or colonization of new host cells. We noted that presence of NFUapi results in more efficient release of liver stage merozoites via merosomes from cultured hepatoma cells. This effect coincides with the increased expression of *Plasmodium yoelii NFUapi* in late liver stages [25]. Interestingly, the bird-infecting malaria parasite *Plasmodium gallinaceum* appears to lack a gene encoding NFUapi, while all SUF elements were readily identified (data not shown). Though this might be easily explained by incomplete sequence data available for this *Plasmodium* species, it is striking that another gene encoding a *Plasmodium*-specific apicoplast protein important for liver merozoite formation, termed PALM, is also missing from its genome [26].

During the course of our studies, we made the observation that fosmidomycin treatment does not impair apicoplast biogenesis or morphology during liver stage maturation. A previous report by Nair and colleagues reported a remarkable inhibition of intra-hepatic parasite growth in the presence of 10 µM fosmidomycin [14]. Using our *nfu::tag* line, we show that upon addition of up to 100 µM fosmidomycin, liver stages are indistinguishable from untreated cultured cells and grow perfectly normal. This finding corroborates our own previous data [15], where we found that fosmidomycin is inactive against *Plasmodium* liver stages. While we cannot conclusively solve this apparent discrepancy, we emphasize that alternative approaches, including facilitated uptake of fosmidomycin to host cells, will eventually help addressing this important topic. Fosmidomycin was extensively tested in phase II clinical trials against malaria [27]–[32]. Although evidence for causal-prophylactic activity of fosmidomycin is limited to this one report [14] and not supported by clinical data, further studies to improve and potentially expand fosmidomycin efficacy to life cycle stages other than asexual *Plasmodium* blood stages are highly desirable.

In conclusion, our data provide experimental evidence that NFUapi localizes to the *Plasmodium* apicoplast and may play specialized and/or redundant roles in the [Fe-S] biosynthetic pathway.

## Materials and Methods

### Ethics Statement

This study was carried out in strict accordance with the German ‘Tierschutzgesetz in der Fassung vom 22. Juli 2009’ and the Directive 2010/63/EU of the European Parliament and Council ‘On the protection of animals used for scientific purposes’. The protocol was approved by the ethics committee of the Berlin state authority (‘Landesamt für Gesundheit und Soziales Berlin’, permit number G0469/09).

### Experimental Animals, Parasites, and Cell Lines

Female NMRI and C57BL/6 mice were purchased from Charles River Laboratories (Sulzfeld, Germany). C57BL/6 mice were used for sporozoite infections. All other parasite infections were conducted with NMRI mice. Experimental genetics were all performed in *P. berghei* strain ANKA (WT), as control lines the GFPcon [33] (mosquito and liver stage development) or Bergreen [34] (mosquito, liver, and blood stage development) lines were used. *In vitro* liver stage parasite development was analyzed using cultured HuH7 hepatoma cells.

### Bioinformatics and Phylogenetic Analysis

Targeting sequences and putative subcellular localization to the apicoplast or alternatively the mitochondrion were analyzed using ApicoAP [35], PlasmoAP [36], PlasMit [37], and MitoProtII [38].

To investigate phylogenetic relationships between NFU proteins, we collected sequences of 68 NifU-like domain containing proteins from apicomplexan parasites, kinetoplastids, plants, fungi, and animals, combining published data and information available through online databases (Table S1 in File S1). NifU-like domains were identified using Pfam (http://pfam.sanger.ac.uk/) and aligned using ClustalW2 and T-Coffee (http://www.ebi.ac.uk/Tools/msa/) using default settings. Calculations of both protein parsimonies and maximum likelihood distances of both alignments resulted in the same overall tree topology. For the tree displayed in Fig. 2, maximum likelihood distances were calculated from 100 bootstrap trees (each with 10x jumbling) of the ClustalW2 alignment with *Azotobacter vinelandii* NifU as the outgroup using programs of the PHYLIP package [39].

### Generation of *NFUapi* Deletion Plasmid and *nfu* ^–^ Parasites

For targeted gene deletion of the *P. berghei NFUapi* gene, fragments of the 5′UTR and of the 3′UTR were amplified from gDNA using gene-specific primers (Table S2 in File S1). PCR fragments were cloned into the *berghei* adaptable transfection vector (pBAT-SIL6) [34], which contains drug-selectable and high-expressing GFP cassettes. First, the 3′UTR homologous sequences were cloned following restriction digestion of vector and insert with HindIII and KpnI. Then, the 5′UTR homologous sequences digested with SacII and EcoRV were cloned into SacII and PvuII linearized vector, thus removing the mCherry-3xMyc tag from the original vector. The resulting plasmids were linearized with SalI and used to transfect *P. berghei* strain ANKA (WT) parasites.

Genotyping of selected parasites was performed by diagnostic PCR using gDNA as template and integration-specific primers (Table S2 in File S1). Integration-specific PCR amplification of the *NFUapi* locus to confirm the predicted deletion of *NFUapi* was done using the following primers: 5′PbHSP70rev and GT-5′NFU-F (5′ integration, 958 bp), and GT-3′NFU-R and 5′PbDHFRrev (3′ integration, 1,210 bp). A clonal *nfu* ^–^ parasite line was generated by intravenous injection of limiting dilutions of parasites into mice. Absence of WT-specific PCR products using primers GT-5′NFU-F and GT-5′NFU-R (5′ WT ANKA control, 960 bp), and GT-3′NFU-F and GT-3′NFU-R (3′ WT ANKA control, 702 bp) (Table S2 in File S1) confirmed the purity of the clonal *nfu* ^–^ parasite line.

### Southern Blot Analysis

The genotype of the clonal *nfu^–^* parasite line was confirmed by Southern blot analysis using the PCR DIG Probe Synthesis kit and the DIG Luminescent Detection kit (Roche), according to the manufacturer’s protocol. For amplification of the hybridization probe, primers TV-3′NFU-F and TV-3′NFU-R (Table S2 in File S1) were used. The hybridization probe was annealed to NdeI digested gDNA resulting in bands of 2.2 kb (WT) and 7.0 kb (*nfu* ^–^).

### Generation of *NFUapi* Tagging Plasmid and *nfu::tag* Parasites

To confirm the apicoplast localization of NFUapi, an mCherry-3xMyc tagged parasite line was generated. For this purpose, the carboxy-terminal part of *NFUapi* was PCR amplified using gene-specific primers (Table S2 in File S1). After restriction digestion with SacII and EcoRV, the fragment was cloned into the SacII and HpaI digested pBAT-SIL6 vector already containing the 3′UTR sequence of *NFUapi*, thus fusing the *NFUapi* carboxy-terminal sequence in frame with the mCherry-3xMyc tag sequence. The resulting plasmid was linearized with SalI and used to transfect *P. berghei* strain ANKA (WT) parasites.

Correct integration by double homologous/ends-out recombination was confirmed by integration-specific PCR using primers GT-C-NFU-F and mCherryRev (5′ integration, 758 bp), and GT-3′NFU-R and 5′PbDHFRrev (3′ integration, 1,210 bp) (Table S2 in File S1). After successful integration, flow cytometric isolation of green fluorescent parasites resulted in an isogenic parasite line [40]. Absence of WT-specific PCR products using primers GT-C-NFU-F and TV-3′NFU-R (5′ WT ANKA control, 1,106 bp), and GT-3′NFU-F and GT-3′NFU-R (3′ WT ANKA control, 702 bp) (Table S2 in File S1) confirmed the purity of the isogenic *nfu::tag* parasite line.

### *Plasmodium* Life Cycle Progression

Gametocyte differentiation and exflagellation of microgametes were examined prior to mosquito feeding. *Anopheles stephensi* mosquitoes were raised under a 14 hour light/10 hour dark cycle, 75% humidity and at 28°C (non-infected) or 20°C (infected), respectively. Sporozoite populations were isolated and analyzed as described previously [41]. Mosquito infectivity was determined at day 10 after feeding. Midgut- and salivary gland-associated sporozoites were quantified at days 14 and 17–21, respectively. To determine sporozoite infectivity, sporozoites were liberated from salivary glands and injected intravenously at the numbers indicated into young, naïve C57BL/6 mice. Patency was determined by daily examination of Giemsa-stained thin blood smears.

*P. berghei in vitro* liver stages were cultured and analyzed using standard techniques [26]. Merosome formation was followed by seeding of 30,000 hepatoma cells per well in eight-well chamber slides (Nalge Nunc International) and inoculation with 10,000 sporozoites 24 hours later. Thereafter, standard procedures for culturing infected hepatoma cells were followed [42]. Merosomes were harvested and counted in a Neubauer chamber 72 hours after infection.

NMRI mice were injected intravenously with 1,000 blood stage Bergreen or *nfu* ^–^ parasites and monitored for blood stage development by daily examination of Giemsa-stained thin blood smears.

### Fluorescence Microscopy

For confirmation of expression and determination of the subcellular localization of NFUapi, fixed *nfu::tag* liver stage parasites were incubated with mouse anti-myc antibodies (1∶1,000 dilution, Santa Cruz Biotechnology) and rabbit anti-*P. berghei* ACP peptide antiserum (1∶750 dilution; [13]). To confirm the apicoplast localization of NFUapi at 48 hours after infection, we treated sporozoite-infected hepatoma cells with 1 µM azithromycin (Pfizer), as described previously [26]. In addition, the effect of 100 µM fosmidomycin (Sigma-Aldrich) on apicoplast morphology during liver stage development was tested. Parasites were identified by staining with rabbit anti-upregulated in infective sporozoites protein 4 (UIS4) peptide antiserum (1∶2,000 dilution; kindly provided by G. Montagna, MPI-IB, Berlin). Branched anti-myc-positive structures extending into the area delineated by the anti-UIS4 antiserum were defined as apicoplasts. Monoclonal mouse anti-*P. berghei* heat shock protein 70 (HSP70) antibodies (1∶300 dilution; [43]) were used to visualize and quantify *nfu* ^–^ liver stage parasites. Bound antibodies were detected using donkey/goat anti-rabbit/mouse IgG Alexa Fluor 488/546 conjugated antibodies (1∶3,000 dilution, Invitrogen). Nuclei were visualized with DNA-dyes Hoechst 33342 (Invitrogen) and DRAQ5 (Axxora; both 1∶1,000 dilution). Coverslips were mounted with Fluoromount-G (Southern Biotech). Total numbers of parasites were counted using a Leica DM2500 epifluorescence microscope. Images were recorded using a Leica TCS SP-1 confocal microscope and processed minimally with ImageJ (http://rsb.info.nih.gov/ij/).

## Supporting Information

File S1 Contains: **Figure S1. Apicomplexan NFUapi proteins.** (A) Primary structure of representative apicomplexan NFUapi proteins. Shown are the overall sequence structures and amino acid sequence identities of NFUapi orthologs in *P. falciparum* (PF3D7_0921400), *P. vivax* (PVX_099490), *Theileria annulata* (TA19885), and *Toxoplasma gondii* (TGME49_021920) compared with *P. berghei* NFUapi (PBANKA_082230). Signal peptide (red), apicoplast-targeting sequence (green), and the NifU-like domain (brown) are shown. (B) Sequence alignment of three *Plasmodium* NFUapi proteins. The NifU-like domain (gray shading) is well conserved. **Table S1.** NifU-like domain containing proteins. **Table S2.** Primer sequences.
(PDF)
