# Supplementary material for: Experimental Genetics of Plasmodium berghei NFU in the Apicoplast Iron-Sulfur Cluster Biogenesis Pathway
Source: PLoS One. 2013 Jun 21;8(6):e67269. doi: 10.1371/journal.pone.0067269 (PMC3689711; doi:10.1371/journal.pone.0067269)
Supplement: File S1 — Contains: Figure S1. Apicomplexan NFUapi proteins. (A) Primary structure of representative apicomplexan NFUapi proteins. Shown are the overall sequence structures and amino acid sequence identities of NFUapi orthologs in P. falciparum (PF3D7_0921400), P. vivax (PVX_099490), Theileria annulata (TA19885), and Toxoplasma gondii (TGME49_021920) compared with P. berghei NFUapi (PBANKA_082230). Signal peptide (red), apicoplast-targeting sequence (green), and the NifU-like domain (brown) are shown. (B) Sequence alignment of three Plasmodium NFUapi proteins. The NifU-like domain (gray shading) is well conserved. Table S1. NifU-like domain containing proteins. Table S2. Primer sequences. (PDF) [file pone.0067269.s001.pdf]

Supplemental information for

Experimental Genetics of *Plasmodium berghei* NFU in the Apicoplast Iron-Sulfur Cluster Biogenesis Pathway

Joana M. Haussig, Kai Matuschewski, Taco W.A. Kooij

Contents:

- **Supplemental Figure S1**
- **Supplemental Table S1 and S2**

Haussig *et al*., Figure S1

A

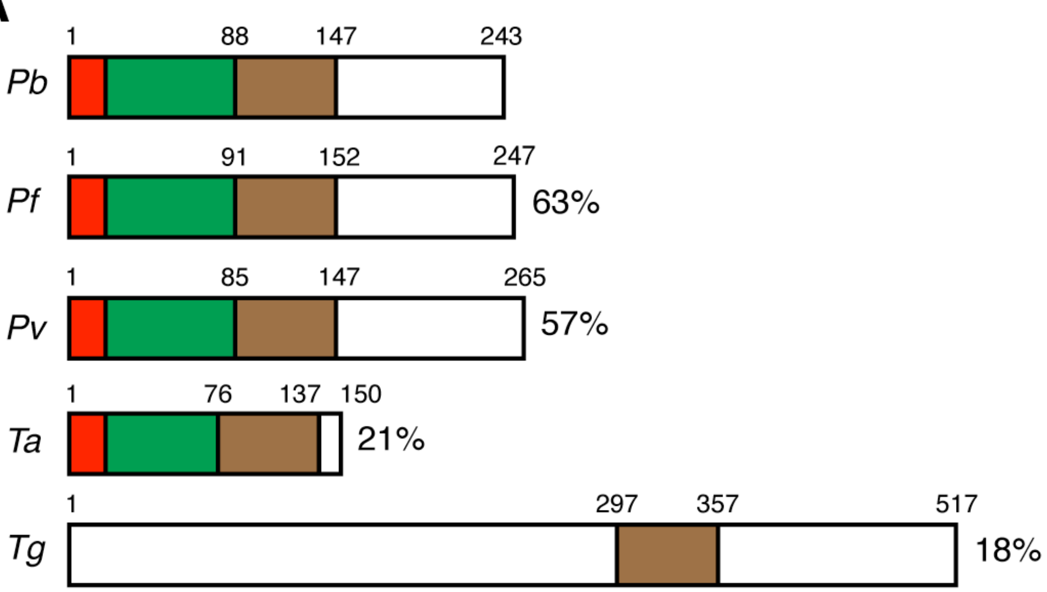

B

```
Pb    MKYSIASMKVTLFFYFLCAKNIASLKYTKSNLSFLYNGCDNINSG----GVNAHRNKT-T
Pf    MNCIFLSVLFIWYFFFVYVKNLSILQYDKAKVLFIQNNFEPLNNNMLRVPLNILRKRTYV
Pv    MLAGSFTLLLSL---FLCAKNAGSLKHPQAALLFVHNSSDLFQND----TLGVLRNKT-K
      *     :: .     *: .** . *:: :: : *: *. : ::..     :.  *:.*

Pb    VQTKNKHSKLKILSLNEKQ-DEELHYELNPENTEKVLNLIRPKLQIDNGDVELVDIKGND
Pf    VKNKKNGVPFQIFLSSEN--DEGLYYELNPENVEKVLNLIRPKLQIDNGDVELVDIKNND
Pv    IQNKKKISRFKIQSSEERHTDGGLHYDLTPENVEKVLNLIRPKLQIDNGDVELVDIKNND
      ::.*::   ::*   .*.  *  *:*:*.***.************************.**

Pb    LYIRLLGNCITCSSNSVTVSQVIKKTLKMYIRGPGNKEPNVIIINFDEINEENILNCLSD
Pf    LYIRLLGNCVTCSSNSITVSHVIKKTLKMYIRNEQNQEPNVIITNFDEINEQNIQNCLSQ
Pv    LYIKLLGNCVTCSSNSVTVSQVIKKTLKMYIRNEKNEEPNVIITNFDEINEENIHSCLSD
      ***.*****:******:***:***********.  *:****** *******:** .***:

Pb    LKPYFDFLKIKLVIKEMINNKENINNSVMLVFKNIENED---------------------
Pf    LKPYLDFLKVEVIIKELVNNKENINNYVCLKFLNIENSS---------------------
Pv    LKPYFDFLKVKVVITDLMKNKENINNSVSLVFKKADLEGGSDADGEADVEAGRSADPYAD
      ****:****::::*.::::******* * * * : : ..

Pb    ---KEVNIPHNIKTEITGRLKQNFPTLTVNFEN
Pf    ---EEINIPHNVKNEITERLKQKFPTLTVNFEN
Pv    PDAEPVKIPFSVKSEITERLKQKFPTLTVNFEN
         :  ::**..:*.*** ****:**********
```

Figure S1. Apicomplexan NFUapi proteins. (A) Primary structure of representative apicomplexan NFUapi proteins. Shown are the overall sequence structures and amino acid sequence identities of NFUapi orthologs in *P. falciparum* (PF3D7_0921400), *P. vivax* (PVX_099490), *Theileria annulata* (TA19885), and *Toxoplasma gondii* (TGME49_021920) compared with *P. berghei* NFUapi (PBANKA_082230). Signal peptide (red), apicoplast-targeting sequence (green), and the NifU-like domain (brown) are shown. (B) Sequence alignment of three *Plasmodium* NFUapi proteins. The NifU-like domain (gray shading) is well conserved.

Table S1. NifU-like domain containing proteins.

Species	Gene Name	Gene ID	Source
APICOMPLEXA			
Plasmodium berghei	*Pb*NFUapi	PBANKA_082230	PlasmoDB
	*Pb*NFU1	PBANKA_083170	PlasmoDB
	*Pb*ISU	PBANKA_131820	PlasmoDB
Plasmodium chabaudi	*Pc*NFUapi	PCHAS_082260	PlasmoDB
	*Pc*NFU1	PCHAS_083200	PlasmoDB
	*Pc*ISU	PCHAS_132150	PlasmoDB
Plasmodium yoelii	*Py*NFUapi	PY02489	PlasmoDB
	*Py*NFU1	PY06884	PlasmoDB
	*Py*ISU	PY00856	PlasmoDB
Plasmodium knowlesi	*Pk*NFUapi	PKH_071860	PlasmoDB
	*Pk*NFU1	PKH_072920	PlasmoDB
	*Pk*ISU	PKH_125200	PlasmoDB
Plasmodium vivax	*Pv*NFUapi	PVX_099490	PlasmoDB
	*Pv*NFU1	PVX_087160	PlasmoDB
	*Pv*ISU	PVX_117755	PlasmoDB
Plasmodium falciparum	*Pf*NFUapi	PF3D7_0921400	PlasmoDB
	*Pf*NFU1	PF3D7_0930900	PlasmoDB
	*Pf*ISU	PF3D7_1454500	PlasmoDB
Babesia bovis	*Bb*NFU1	BBOV_III006970	PiroplasmaDB
	*Bb*ISU	BBOV_IV004050	PiroplasmaDB
Theileria annulata	*Ta*NFUapi	TA19885	PiroplasmaDB
	*Ta*NFU1	TA10900	PiroplasmaDB
	*Ta*ISU	TA19345	PiroplasmaDB
Theileria parva	*Tp*NFUapi	TP01_0062	PiroplasmaDB
	*Tp*NFU1	TP04_0610	PiroplasmaDB
	*Tp*ISU	TP01_0192	PiroplasmaDB
Cryptosporidium muris	*Cm*ISU	CMU_027160	CryptoDB
Cryptosporidium parvum	*Cp*ISU	cgd6_1050	CryptoDB
Eimeria tenella	*Et*NFUapi	ETH_00036850 + ETH_00021275	ToxoDB
	*Et*NFU1	ETH_00027735	ToxoDB
	*Et*ISU	ETH_00024750	ToxoDB
Neospora caninum	*Nc*NFUapi	NCLIV_005330	ToxoDB
	*Nc*NFU1	NCLIV_013030	ToxoDB
	*Nc*ISU	NCLIV_051300	ToxoDB
Toxoplasma gondii	*Tg*NFUapi	TGME49_021920	ToxoDB
	*Tg*NFU1	TGME49_012930	ToxoDB
	*Tg*ISU	TGME49_037560	ToxoDB

Species	Gene Name	Gene ID	Source
KINETOPLASTIDA			
Leishmania major	*Lm*NFU1	LmjF.26.0190	TriTrypDB
	*Lm*NFU2	LmjF.36.0880	TriTrypDB
	*Lm*ISU	LmjF.35.3620	TriTrypDB
Trypanosoma brucei	*Tb*NFU1	Tbg972.7.1730	TriTrypDB
	*Tb*NFU2	Tbg972.10.6450	TriTrypDB
	*Tb*ISU	Tbg972.9.6980	TriTrypDB
Trypanosoma cruzi	*Tc*NFU1	Tc00.1047053508207.130	TriTrypDB
	*Tc*NFU2	Tc00.1047053506679.60	TriTrypDB
	*Tc*ISU	Tc00.1047053507009.90	TriTrypDB
PLANTAE			
Chlamydomonas reinhardtii	*Cr*NIFU1	XP_001695148	GenBank
	*Cr*NIFU2	XP_001690805	GenBank
	*Cr*NIFU3	XP_001691526	GenBank
	*Cr*ISU1	XP_001693712	GenBank
Arabidopsis thaliana	*At*NFU1	NP_567219	GenBank
	*At*NFU2	NP_568715.1	GenBank
	*At*NFU3	NP_567735	GenBank
	*At*NFU4	NP_566673	GenBank
	*At*NFU5	NP_175550	GenBank
	*At*ISU1	NP_193953	GenBank
	*At*ISU2	NP_186751	GenBank
	*At*ISU3	NP_192317	GenBank
FUNGI			
Cryptococcus neoformans	*Cn*Nfu_N	XP_571879	GenBank
	*Cn*IscU	XP_569269	GenBank
Saccharomyces cerevisiae	*Sc*Nfu1	NP_012884	GenBank
	*Sc*Isu1	NP_015190	GenBank
	*Sc*Isu2	NP_014869	GenBank
ANIMALIA			
Mus musculus	*Mm*Nfu1	NP_001164062	GenBank
	*Mm*Iscu	NP_079802	GenBank
Homo sapiens	*Hs*NFU1	NP_056515	GenBank
	*Hs*ISCU	NP_055116	GenBank
BACTERIA			
Azotobacter vinelandii	*Av*NifU	YP_002797400	GenBank

Table S2. Primer sequences.

Primer Name[a]	Primer Sequence (REase sites underlined)	REase	Size WT (bp)[b]	Size INT (bp)[c]
5'PbHSP70rev	CAATTTGTTGTACATAAAATAGGCAG			
5'PbDHFRrev	ATGAAATACCGCTCCATTTTTCC			
mCherryRev	CCCTCCATGTGAACCTTGAAG			
TV-5'NFU-F	TATCCGCGGTTTTTCCTATAAATGTGTGTGTAATGC	SacII	599	
TV-5'NFU-R	AAAGATATCACTCACAAAAATAAGCACATGATTG	EcoRV		
TV-C-NFU-F	AAACCGCGGCGTGTAGCTCAAATAGTGTAACTG	SacII	576	
TV-C-NFU-R	TAAGATATCATTTTCAAAATTAACAGTTAATGTTGGAAA	EcoRV		
TV-3'NFU-F	TATAAGCTTCAGCGGCTAGTCAATCTAATC	HindIII	439	
TV-3'NFU-R	TAAGGTACCGTTCATTTAAAACACCAGCGAG	KpnI		
GT-5'NFU-F	ATTTTTTCTCTGGTTTTCATTTATATTTC		960	958
GT-5'NFU-R	TTGTTTTATTTCTGTGTGCATTAACC			
GT-C-NFU-F	GCCCCAAATTACAAATAGATAATGG		1106[d]	758[d]
GT-3'NFU-F	GAAAATGAAGATAAGGAAGTAAATATACC		702	
GT-3'NFU-R	TAATATACATAAATGTTATTGGGATCTTTTG			1210

[a] TV and GT indicate primers designed for the generation of Transfection Vectors and GenoTyping.
[b] Sizes of the PCR products of forward (F) and reverse (R) primers on WT gDNA.
[c] Sizes of the respective integration-specific PCR products; forward 5' gene-specific primers combined with 5'PbHSP70rev and reverse 3' gene-specific primers combined with 5'PbDHFRrev.
[d] WT-specific reaction with TV-3'NFU-R; integration-specific reaction with mCherryRev.
